# Dramatic mass loss in extreme high-elevation areas of a western Himalayan glacier: observations and modeling

**DOI:** 10.1038/srep30706

**Published:** 2016-08-26

**Authors:** Huabiao Zhao, Wei Yang, Tandong Yao, Lide Tian, Baiqing Xu

**Affiliations:** 1 Key Laboratory of Tibetan Environment Changes and Land Surface Processes, Institute of Tibetan Plateau Research, Chinese Academy of Sciences (CAS), Beijing 100101, China; 2 CAS Center for Excellence in Tibetan Plateau Earth Sciences, Beijing 100101, China

## Abstract

Rapid climate change at high elevations has accelerated glacier retreat in the Himalayas and Tibetan Plateau. However, due to the lack of long-term glaciological measurements, there are still uncertainties regarding when the mass loss began and what the magnitude of mass loss is at such high elevations. Based on *in situ* glaciological observations during the past 9 years and a temperature-index mass balance model, this study investigates recent mass loss of the Naimona’nyi Glacier in the western Himalayas and reconstructs a 41-year (1973/74–2013/14) equilibrium line altitude (ELA) and glacier-wide mass loss. The result indicates that even at 6000 m above sea level (a.s.l.), the annual mass loss reaches ~0.73 m water equivalent (w.e.) during the past 9 years. Concordant with the abrupt climate shift in the end of 1980s, the ELA has dramatically risen from ~5969 ± 73 m a.s.l. during 1973/74–1988/89 to ~6193 ± 75 m a.s.l. during 1989/90–2013/14, suggesting that future ice cores containing uninterrupted climate records could only be recovered at least above 6200 m a.s.l. in the Naimona’nyi region. The glacier-wide mass balance over the past 41 years is averaged to be approximately −0.40 ± 0.17 m w.e., exhibiting a significant increase in the decadal average from −0.01 ± 0.15 to −0.69 ± 0.21 m w.e.

Mass loss of Himalayan glaciers is of broad societal concern due to the possible impact on water supplies in Asia^1^,^2^. With recent rapid climate warming^3^,^4^, most of the Himalayan glaciers have suffered from negative mass balance and have exhibited accelerated mass loss and area reduction during the past decades^5^,^6^,^7^,^8^,^9^,^10^.

Among all of the shrinking Himalayan glaciers, the highest elevation where net mass loss occurred was in the western Himalayas. The lack of radiogenic bomb horizons in the ice cores from the Naimona’nyi Glacier (30°27′N, 81°92′E, 6050 m a.s.l.) suggests no net mass accumulation at the site since at least the 1950s in western Himalayas^11^. Geodetic studies, including ICESat and differential GPS measurements, confirmed the recent ice thinning above 6000 m a.s.l. at the Naimona’nyi Glacier^12^,^13^,^14^,^15^. These data reveal that the multi-year averaged ELA could possibly increase above 6000 m a.s.l. during some periods in the past decades. In contrast, both the measured ELAs and firn line altitudes in the surrounding Himalayas did not break this record kept by the Naimona’nyi Glacier^16^,^17^,^18^. Anomalous ELAs above 6000 m a.s.l. have never been reported in the Glacier Mass Balance Bulletin compiled by the World Glacier Monitoring Service^19^. The surface mass loss in extreme high-elevation areas of the glacier in the western Himalayas indicates rapid changes in hydrothermal conditions in the troposphere.

However, the lack of long-term glaciological measurements limits our understanding of when surface mass loss began above 6000 m a.s.l. and the overall magnitude of mass balance at the Naimona’nyi Glacier during the past decades. In addition, it also prevents us from identifying the minimum elevation above which successive, high-quality paleoclimatic records can be retrieved for this critical region that is influenced by the South Asian summer monsoon and the Westerlies^11^,^20^. In attempt to fill these gaps, a glaciological measurement campaign was carried out at the Naimona’nyi Glacier over the past decade by the Institute of Tibetan Plateau Research, Chinese Academy of Sciences. Based on these *in situ* measurements and a temperature-index mass balance model, here we investigate the magnitude of and temporal changes to the surface mass losses of the Naimona’nyi Glacier during the past four decades (1973/74–2013/14) in an effort to improve our knowledge of glacier status in the extreme high elevation area of the western Himalayas.

The Naimona’nyi Glacier is located in the western Himalayas in the southwestern Tibetan Plateau (Fig. 1a). This glacier is one of the largest glaciers surrounding the Naimona’nyi peak (7694 m a.s.l.) and has a total area of 14.4 km^2^ with two branches flowing both northward and southeastward (Fig. 1b). More than 90% of the area is below 6200 m a.s.l., with half of the area concentrated in the altitudinal zone of 6000 to 6200 m a.s.l. (Fig. 1c), including a shallow sloping ice field where ice cores were drilled at 6050 m a.s.l. in 2006^11^,^20^. The maximum thickness of this ice field exceeds 250 m^12^. Regional glacier area in the Naimona’nyi region has decreased by approximately 8% between 1976 and 2003^21^, and the regional mass loss rate was estimated to be between −0.37 ± 0.25 and −0.43 ± 0.09 m w.e. a^−1^ during the period from 2003 to 2009^8^,^9^.

## Results and Discussion

### Observed and modeled mass loss of the Naimona’nyi Glacier

Although stake measurements display significant spatial differences even at the same elevations due to the topographical effect (e.g., aspect, shading, snow drift), such data are helpful for deriving the mean mass loss as a function of elevation during the past decade (Fig. 2). The annual mass balance gradient between 5800 to 6100 m a.s.l. ranged from ~0.25 to ~0.38 m w.e. (100 m)^−1^. From point measurements and mass balance gradients, it is calculated that the ELA during six out of nine years was above the elevation of the highest measuring stake (i.e., 6100 m a.s.l.). The two lowest ELAs were estimated to be ~6080 m a.s.l. and ~6060 m a.s.l. for the balance years 2009/10 and 2012/13, respectively. Mass loss at 6000 m a.s.l. ranged from a minimum of 0.23 m w.e. (2012/13) to a maximum of 1.33 m w.e. (2013/14), with average mass loss of ~0.73 m w.e. a^−1^ for the entire time span.

The modeling performance of annual mass balance as a function of elevations is shown in Fig. 2. The grey lines envelop the first 100 optimized results among 1500 random simulations, with a root mean square difference (RMSD) less than 0.5 m w.e. a^−1^ between the measured and simulated values at the stakes (Supplementary Figs S1 and S2). Because many factors (e.g., topographic shading, snow drift) can contribute to the spatial heterogeneity of mass loss, the overall agreement between measurements and model simulations demonstrates both the effectiveness of mass balance reconstruction using the simply mass-balance model and its feasibility for evaluating glacier status during the past four decades. Thus, the 41-year ELA and glacier-wide mass balances were reconstructed (Fig. 3a,b). The modeled glacier-wide mass balances are compared with the geodetic mass balances in different periods^14^,^15^, showing good agreement between the modeled values of −0.32 ± 0.19 and −0.63 ± 0.20 m w.e. and the geodetic values of −0.40 ± 0.29 and −0.54 ± 0.28 m w.e. for 1974–2006 and 2000–2009, respectively (Fig. 3b). The average annual mass balance during the past four decades was −0.40 ± 0.17 m w.e., with decadal averages of −0.01 ± 0.15 m w.e. for 1973/74–1982/83, −0.32 ± 0.16 m w.e. for 1983/84–1992/93, −0.58 ± 0.19 m w.e. for 1993/94–2002/03 and −0.69 ± 0.21 m w.e. for 2003/04–2013/14. Negative mass balances are observed in most years over the past four decades, indicating the continuous mass deficit of the Naimona’nyi Glacier since the 1970s.

### Mass loss above 6000 m a.s.l

The ELA reconstruction can be used to determine when the surface mass loss above 6000 m a.s.l. began and from where successive, high-quality paleoclimatic records can be retrieved. The mean ELA stays at ~5935 ± 76 m a.s.l. during the first decade (1973/74–1982/83), increases to ~6080 ± 74 m a.s.l. in 1983/84–1992/93 and ~6184 ± 74 m a.s.l. in 1993/94–2002/03, and finally reaches ~6212 ± 77 m a.s.l. in 2003/04–2013/14. (Fig. 3a). The decadal increase in the ELA during the past four decades was approximately 280 m. The maximum ELA was ~6365 m a.s.l. in 2006/07 and the minimum was ~5668 m a.s.l. occurred in 1974/75.

To further illustrate the temporal changes in surface mass loss at above 6000 m a.s.l., the accumulative mass balances from the two different altitudes (6060 and 6220 m a.s.l.) were generated (Fig. 3c,d). At the elevation closer to the ice core drilling site in 2006^11^, there was only a small mass accumulation during the period from 1973/74 to the late-1980s (Fig. 3c). However, after that, there was continuous negative mass balance resulting in a total mass loss of ~4.7 m w.e. by 2013/14. The distinctive characteristics are portrayed by the less magnitude of mass gain during 1973/74-1988/89 but an accelerating mass loss trend after that period. As a comparison, the ELA increases largely from ~5969 ± 73 m a.s.l. to 6193 ± 75 m a.s.l. with a dramatic enhancement of glacier-wide mass loss from −0.06 ± 0.15 to −0.62 ± 0.19 m w.e. The turning point corresponds to an abrupt climate change recorded at the Burang station (30°17′N, 81°15′E, 3900 m a.s.l., 1973–2014), including a dramatic temperature increase and significant precipitation decrease in the late-1980s (Supplementary Fig. S3). The positive accumulations at 6220 ma.s.l. from 1973/74 to 2013/14 (Fig. 3d) likely indicates that the lower limit at which ice cores can provide successive, high-quality paleoclimatic records in the Naimona’nyi region. Similarly to this region, a few ice cores drilled in the southern and central Tibetan Plateau^22^ also found no net accumulation even at 5800 m a.s.l. This suggests that the area of accumulation is becoming a ablation zone with the dramatic rise in ELA^22^. However, due to few *in-situ* glacio-meteorological observations, it is hard to precisely identify the ideal elevations for ice core drilling in these regions. Thus, more glaciological studies should be carried out to detect mass loss in extreme high elevations.

Due to the lack of long-term meteorological data before 1973 at the Burang station and in surrounding regions, the grid data of temperature and precipitation from the CRU TS 3.22 dataset^23^ covering the Burang station were used to evaluate the climate background before 1973 (Supplementary Fig. S4). As expected, compared to the period of the 1970s and 1980s, the Naimona’nyi region was under relatively warm, dry conditions between the 1940s and 1960s. Based on the variations in temperature and precipitation, it can be speculated that the mean ELA during the 1940s–1960s might have been higher than the mean value of ~5935 ± 76 m a.s.l. between 1973/74 and 1982/83, but should be much lower than that of ~6212 ± 77 m a.s.l. over the last decade. Thus, it is possible that mass loss above 6000 m a.s.l. on the Naimona’nyi Glacier has occurred since at least the 1940s. In this context, the fallout radionuclides from atmospheric thermonuclear bomb tests in the 1950s and 1960s were not contained initially in the ice body at the elevation band 6025–6200 m a.s.l. of this glacier. This finding does not provide a definitive conclusion, however, because the lack of radionuclides could be due to lack of accumulation in the 1950s and 1960s or subsequent mass loss of ice containing these particles.

The mass loss above 6000 m a.s.l. on the Naimona’nyi Glacier in the western Himalayas can be attributed to unique geographical and topographical conditions. Generally, annual precipitation has a negative gradient from east to west on the Tibetan Plateau^24^. In addition, the Himalayan range acts as an effective orographic precipitation barrier and creates a strong rain lee-effect for the regions north of the range, with drier air masses and less precipitation^25^. The mean annual precipitation was only 157 mm at the Burang meteorological station during1973–2014. Even at AWS2 (5950 m a.s.l), only 221 mm of precipitation was recorded by a T-200B precipitation gauge during October 2013 to September 2014. From the perspective of energy and mass balance, precipitation contributes to mass accumulation while solar radiation promotes glacier melt. In the Naimona’nyi region, on one hand, less precipitation positively limits the glacier accumulation; on the other hand, more solar radiation energy related to the low latitude and atmosheric moisture content could arrive and possibly be absorbed for snow and ice melting at the glacier surface by an accumulation-albedo-melt feedback mechanism^26^. Furthermore, the increased air tempeature in recent decades (Supplementary Figs S3 and S4) has also contributed much energy for glacier mass loss in higher elevations. As a result, the enhanced energy supply with less precipitation for glaciers on the lee side of western Himalayas has caused some of the highest ELAs and net mass losses at high elevations worldwide.

## Summary

In this study, our *in situ* measurements show that even at 6000 m a.s.l., the mean mass loss of the Naimona’nyi Glacier reached as much as ~0.73 m w.e. a^−1^ over the past 9 years. Using a calibrated mass balance model, the variations in mass loss and ELA over the past four decades were reconstructed and analyzed. We found that annual averaged mass loss of the Naimona’nyi Glacier had increased over this time period from ~0.01 ± 0.15 m w.e. (1973/74–1982/83) to ~0.69 ± 0.21 m w.e. (2003/04–2013/14). We also observed accelerating trends of mass loss and ELA rise beginning in the late-1980s, which are associated with a shift in regional climate condition. The ELA has risen approximately 280 m in the previous four decades and reaching an average elevation of ~6212 ± 77 m in the last decade. According to the regional climate condition, the net mass loss on the Naimona’nyi Glacier above 6000 m a.s.l. has been possibly occurring since as early as the 1940s.

Our study also sheds light on the possible minimum elevation at which future ice cores can be drilled to obtain successive, high-quality paleoclimatic records in the north western Himalayas. Concordant with a recent report on the mass loss at high elevations of southern and central Tibetan Plateau inferred from ice core records^22^, there is an urgent need to recover more ice cores from the Tibetan Plateau and the surrounding region; this area is a key region in climate research and such data must be collected before disappearance of the glacial records preserved therein.

## Materials and Methods

### Observations

**Figure 1:**
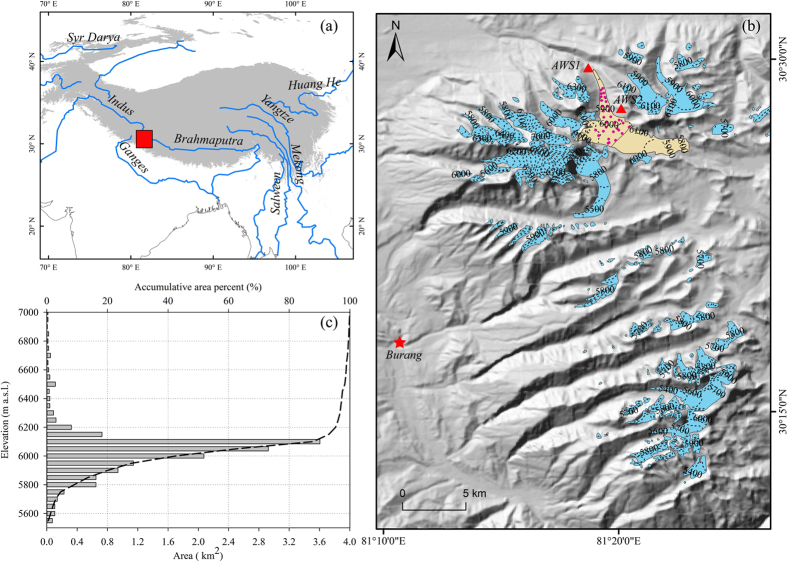
The Naimona’nyi Glacier. (**a**) location of Naimona’nyi Glacier in the southwestern Tibetan Plateau (red rectangle) at the head of both the Brahmaputra and Indus Rivers; (**b**) a topographical map with the distribution of measuring stakes in 2013 on the Naimona’nyi Glacier (pink dots), the AWS sites (red triangles), and the location of Burang station (red star); (**c**) area-elevation distribution (grey bar) and accumulative area percent (dash line) of the Naimona’nyi Glacier. The grey shading in (**a**) denotes the area with an elevation above 2500 m. Maps (**a**,**b**) were made using ArcGIS v9.3 (www.esri.com).

**Figure 2:**
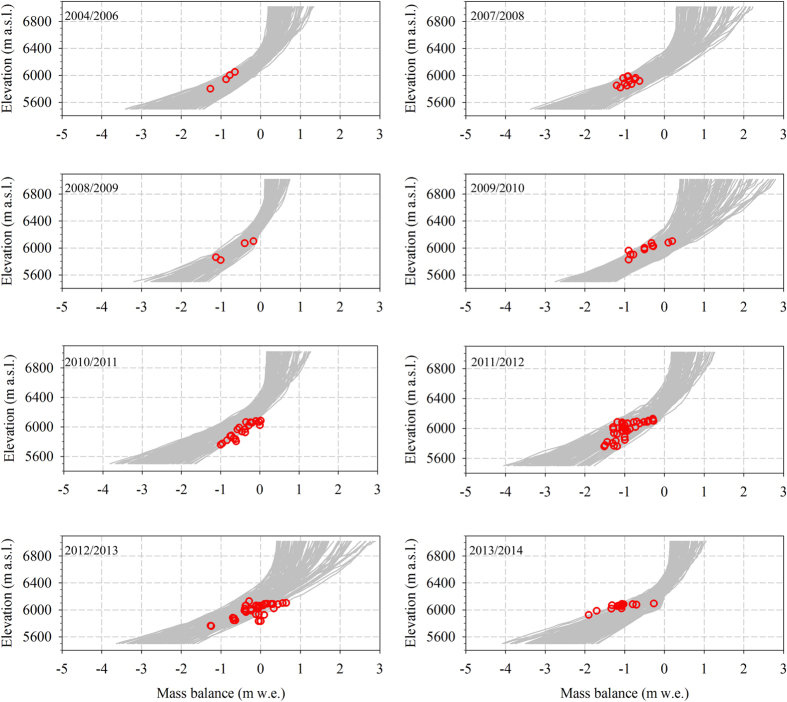
Comparison between modeled (grey curves) and measured (red circles) annual mass balance as a function of elevation on the Naimona’nyi Glacier during the past nine balance years. Note that the period of 2004 to 2006 covers two mass balance years.

**Figure 3:**
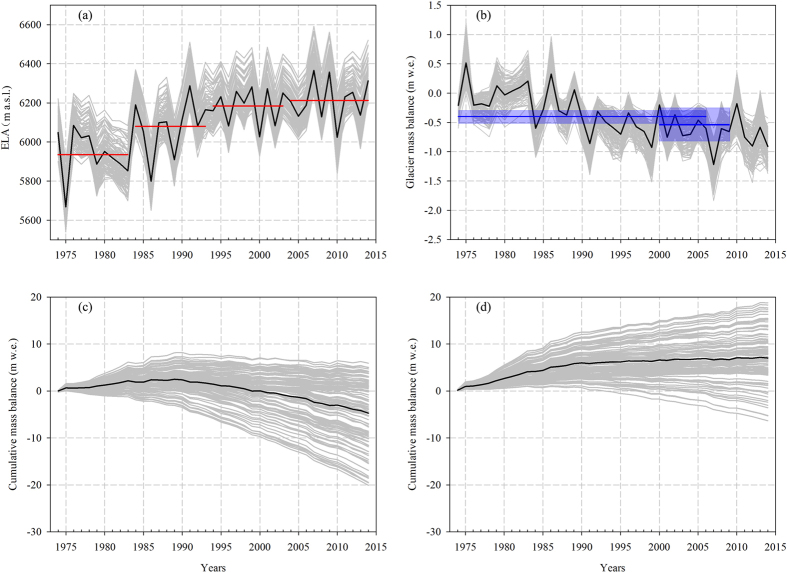
(**a**) Fluctuation of ELAs on the Naimona’nyi Glacier for the past four decades, with the decadal averages since 1974 (red lines); (**b**) Temporal changes in area-averaged mass balance, including a comparison with geodetic results^14^,^15^ (blue lines); (**c**,**d**) Cumulative mass balance for elevations of 6060 m a.s.l. and 6220 m a.s.l., respectively.

Measurements of glacier mass balance were made using the glaciological method on the northern branch of the Naimona’nyi Glacier beginning in 2004 (Fig. 1b). The number of monitoring stakes over the entire glacier surface has progressively increased from four in 2004 to 31 in 2013. The heights of the individual stakes and measurements of snow pits were manually recorded in early October of every year to derive the annual mass balance. Due to logistical issues, no fieldwork was carried out in 2005, and the stakes drilled in 2004 were missing when the sites were revisited in October 2007. The annual mass balances for nine balance years, including the average value during the 2004–06 balance year, are available for this glacier over the past decade (Fig. 2). The temperature and precipitation used to force the mass balance model was recorded at the nearest meteorological station (Burang station), which is located approximately 20 km from the Naimona’nyi Glacier (Fig. 1).

### Mass-balance model

A simple temperature-index mass balance model was used in this study. The mass balance, *B(i, t)*, in *i*^*th*^ different elevations (*z*_*i*_: an interval of 40 m) on day *t* was calculated using the tempeature-index melting model^27^ and an accumulation model:


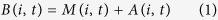



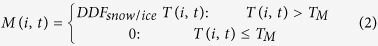



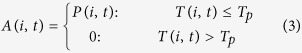



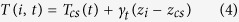



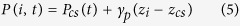


Where *M(i, t)* is the daily melting (mm w.e.) and *A(i, t)* is the daily accumulation (mm w.e.) in *i*^*th*^ elevations; *DDF*_*snow/ice*_ is the melting factor for snow or ice; *T(i, t)* and *P(i, t)* are the daily mean air temperature and daily precipitation, respectively, which are extrapolated from the Burang meteorological station (*T*_*cs*_ and *P*_*cs*_) using the variable yearly temperature lapse rate (*γ*_*t*_) and a constant precipitation gradient (*γ*_*p*_); *z*_*cs*_ and z_*i*_ are the elevation of Burang station and the different elevational bands, respectively, and *T*_*M*_/*T*_*p*_ are the threshold temperatures below or above which melt or solid snowfall is assumed to be zero. The area-averaged mass balance (*B*_*n*_) is calculated by considering the area weight at each elevation bin. Based on the topographic map compiled in 1976 and two Landsat satellite images taken in 1999 and 2007, the temporal change of glacier area was considered in the model by linear area interpolation^28^.

In the mass-balance model, the annual *γ*_*t*_ was calculated using ERA Interim reanalysis temperature data (June-September, 1979–2014) from 3 × 3 grids around the Naimona’nyi Glacier^29^. The *γ*_*t*_ for the period from 1974 to 1978 is assumed to be same as the value in 1979. The temperature bias was corrected by comparison with temperature records at AWS1 in 5500 m a.s.l. (Supplementary Fig. S5). In the model, five parameters were first calibrated and evaluated to determine the introduction of uncertainies^30^,^31^,^32^ (Supplementary Table S1). A Monte Carlo simulation consisting of 1500 realizations was performed to estimate the model uncertainties^33^,^34^. In this experiment, a pool of 100 out of the 1500 parameter combinations yielded the RMSD less than 0.50 m w.e. between the modeled and measured mass balance on the surface of the Naimona’nyi Glacier from 2004 to 2014 (Supplementary Fig. S1 and Fig. 2). Simulations with these 100 parameter combinations were selected for this study. The 100 individidual modeling outcomes represent the possible uncertainty ranges, and the average value was adopted as the accepted value for the Naimona’nyi Glacier.

## Additional Information

**How to cite this article**: Zhao, H. *et al*. Dramatic mass loss in extreme high-elevation areas of a western Himalayan glacier: observations and modeling. *Sci. Rep.* **6**, 30706; doi: 10.1038/srep30706 (2016).

## Supplementary Material

Supplementary Information

## References

[b1] Immerzeel W. W., van Beek L. P. H. & Bierkens M. F. P. Climate change will affect the Asian water towers. Science 328, 1382–1385 (2010). 20538947 10.1126/science.1183188

[b2] Kaser G., Großhauser M. & Marzeion B. Contribution potential of glaciers to water availability in different climate regimes. Proc. Natl. Acad. Sci. USA 107, 20223–20227 (2010). 21059938 10.1073/pnas.1008162107 PMC2996705

[b3] Prasad A. K., Yang K. H. S., El-Askary H. M. & Kafatos M. Melting of major Glaciers in the western Himalayas: evidence of climatic changes from long term MSU derived tropospheric temperature trend (1979–2008). Ann. Geophys. 27, 4505–4519 (2009).

[b4] Yan L. & Liu X. Has climatic warming over the Tibetan Plateau paused or continued in recent Years? J. Earth Ocean Atmos. Sci. 1, 13–28 (2014).

[b5] Azam M. F. . Reconstruction of the annual mass balance of Chhota Shigri glacier, Western Himalaya, India, since 1969. Ann. Glaciol. 55, 69–80 (2014).

[b6] Bolch T. . The state and fate of Himalayan glaciers. Science 336, 310–314 (2012). 22517852 10.1126/science.1215828

[b7] Fujita K. & Nuimura T. Spatially heterogeneous wastage of Himalayan glaciers. Proc. Natl. Acad. Sci. USA 108, 14011–14014 (2011). 21808042 10.1073/pnas.1106242108 PMC3161597

[b8] Kääb A., Treichler D., Nuth C. & Berthier E. Brief communication: Contending estimates of 2003–2008 glacier mass balance over the Pamir–Karakoram–Himalaya. The Cryosphere 9, 557–564 (2015).

[b9] Neckel N., Kropáček J., Bolch T. & Hochschild V. Glacier mass changes on the Tibetan Plateau 2003–2009 derived from ICESat laser altimetry measurements. Environ. Res. Lett. 9, 014009 (2014).

[b10] Yao T. . Different glacier status with atmospheric circulations in Tibetan Plateau and surroundings. Nat. Clim. Change 2, 663–667 (2012).

[b11] Kehrwald N. M. . Mass loss on Himalayan glacier endangers water resources. Geophys. Res. Lett. 35, L22503 (2008).

[b12] Tian L. . Direct measurement of glacier thinning on the southern Tibetan Plateau (Gurenhekou, Kangwure and Naimona’Nyi glaciers). J. Glaciol. 60, 879–888 (2014).

[b13] Zhu D., Tian L., Wang J., Wang Y. & Cui J. Rapid glacier retreat in the Naimona’Nyi region, western Himalayas, between 2003 and 2013. J. Appl. Remote Sens. 8, 083508 (2014).

[b14] Zong J., Ye Q. & Tian L. Recent Naimona’nyi glacier surface elevation changes on the Tibetan Plateau based on ICESat/GLAS, SRTM DEM and GPS measurements. Chinese Sci. Bull. 21, 2108–2118 (2014).

[b15] Zong J. Study of glacier volume changes based on multi-data sources from space and *in-situ* measurements in Mt. Everest and Mt. Naimona’nyi area. PhD thesis. University of Chinese Academy of Sciences (2015).

[b16] Guo Z. . Temporal and spatial changes in Western Himalayan firn line altitudes from 1998 to 2009. Global Planet. Change 118, 97–105 (2014).

[b17] Wagnon P. . Four years of mass balance on Chhota Shigri Glacier, Himachal Pradesh, India, a new benchmark glacier in the western Himalaya. J Glaciol. 53, 603–611 (2007).

[b18] Wagnon P. . Seasonal and annual mass balances of Mera and Pokalde glaciers (Nepal Himalaya) since 2007. The Cryosphere 7, 1769–1786 (2013).

[b19] WGMS. Glacier Mass Balance Bulletin No. *12* (*2010–2011*) (eds Zemp M. . ) 1–106 (World Glacier Monitoring Service, 2013).

[b20] Thompson L. G., Mosley-Thompson E., Davis M. E. & Brecher H. H. Tropical glaciers, recorders and indicators of climate change, are disappearing globally. Ann. Glaciol. 52, 23–34 (2011).

[b21] Ye Q., Yao T., Kang S., Chen F. & Wang J. Glacier variations in the Naimona’nyi region, western Himalaya, in the last three decades. Ann. Glaciol. 43, 385–389 (2006).

[b22] Kang S. . Dramatic loss of glacier accumulation area on the Tibetan Plateau revealed by ice core tritium and mercury records. The Cryosphere 9, 1213–1222 (2015).

[b23] Harris I., Jones P. D., Osborn T. J. & Lister D. H. Updated high-resolution grids of monthly climatic observations – the CRU TS3.10 Dataset. Int. J. Climatol. 34, 623–642 (2014).

[b24] Tong K., Su F., Yang D., Zhang L. & Hao Z. Tibetan Plateau precipitation as depicted by gauge observations, reanalyses and satellite retrievals. Int. J. Climatol. 34, 265–285 (2014).

[b25] Maussion F. . Precipitation seasonality and variability over the Tibetan Plateau as resolved by the high Asia reanalysis. J. Clim. 27, 1910–1927 (2014).

[b26] Mölg T., Maussion F. & Scherer D. Mid-latitude westerlies as a driver of glacier variability in monsoonal High Asia. Nat. Clim. Change 4, 68–73 (2014).

[b27] Hock R. Temperature index melt modelling in mountain areas. J. Hydrol. 282, 104–115 (2003).

[b28] Zemp M. . Reanalysing glacier mass balance measurement series. The Cryosphere 7, 1227–1245 (2013).

[b29] Dee D. . The ERA-Interim reanalysis: Configuration and performance of the data assimilation system. Q. J. Roy. Meteor. Soc. 137, 553–597 (2011).

[b30] Zhang Y., Liu S. & Ding Y. Observed degree-day factors and their spatial variation on glaciers in western China. Ann. Glaciol. 43, 301–306 (2006).

[b31] Braithwaite R. J. Positive degree-day factors for ablation on the Greenland ice sheet studied by energy-balance modelling. Ann. Glaciol. 41, 153–160 (1995).

[b32] Thayyen R. J., Gergan J.T. & Dobhal D. P. Monsoonal control on glacier discharge and hydrograph characteristics, a case study of Dokriani Glacier, Garhwal Himalaya, India. J. Hydrol. 306, 37–49 (2005).

[b33] Mölg T., Maussion F., Yang W. & Scherer D. The footprint of Asian monsoon dynamics in the mass and energy balance of a Tibetan glacier. The Cryosphere 6, 1445–1461 (2012).

[b34] Yang W. . Mass balance of a maritime glacier on the southeast Tibetan Plateau and its climatic sensitivity. J. Geophys. Res. Atmos. 118, 9579–9594 (2013).

