# Supplementary material for: Dramatic mass loss in extreme high-elevation areas of a western Himalayan glacier: observations and modeling
Source: Sci Rep. 2016 Aug 26;6:30706. doi: 10.1038/srep30706 (PMC4999863; doi:10.1038/srep30706)
Supplement: Supplementary Information [file srep30706-s1.docx]

*Supplementary information*

**Dramatic mass loss in extreme high-elevation areas of a western Himalayan glacier: observations and modeling**

Huabiao Zhao^1,2^, Wei Yang^1,2*^, Tandong Yao^1,2^, Lide Tian^1,2^& Baiqing Xu^1,2^

^1^Key Laboratory of Tibetan Environment Changes and Land Surface Processes, Institute of Tibetan Plateau Research, Chinese Academy of Sciences (CAS), Beijing 100101, China

^2^CAS Center for Excellence in Tibetan Plateau Earth Sciences, Beijing 100101, China

^*^Corresponding author: W. Yang, Institute of Tibetan Plateau Research, Chinese Academy of Sciences, Building 3, Courtyard 16, Lincui Road, Chaoyang District, Beijing 100101, China. Email: [yangww@itpcas.ac.cn](mailto:yangww@itpcas.ac.cn)

**Table S1.** The lower bound(LB) and upper bound(UB) of parameters in the mass-balance model.

| Parameters | LB | UB | References |
| --- | --- | --- | --- |
| *DDF_snow_* (mm d^-1 o^C^-1^) | 2 | 6 | ^27,30^ |
| *DDF_ice_*  (mm d^-1 o^C^-1^) | 5 | 15 | ^27,30^ |
| *γ_p_* (% 100m^-1^) | 1 | 30 | This study |
| *T_M_* (^o^C) | -2 | 4 | ^31^ |
| *T_P_* (^o^C) | -2 | 4 | ^32^ |

**
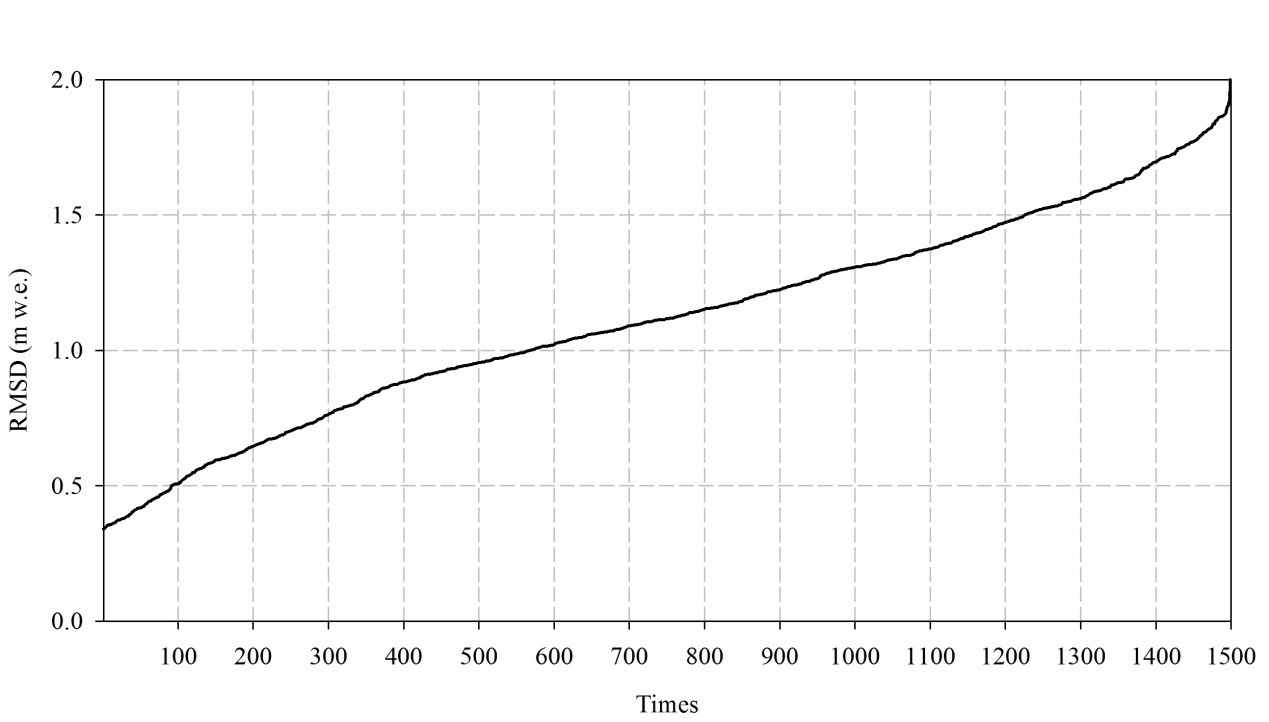
**

**Figue S1.** The root mean square difference (RMSD) under the 1500 Monte Carlo simulations.


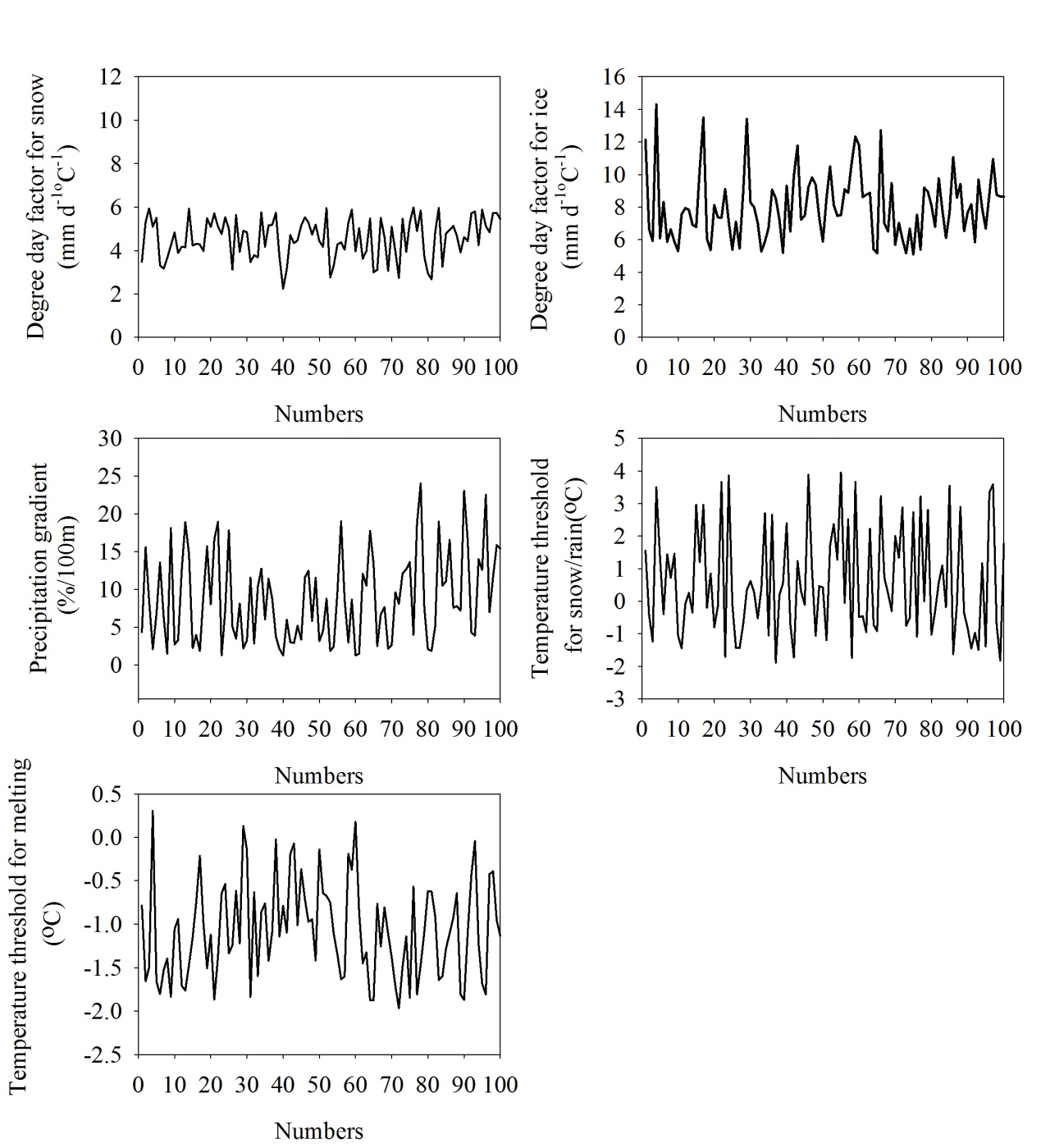


**Figure S2.** The fluctuation of five key parameters in the first 100 optimized parameter combinations selected by the Monte Carlo simulations in the model


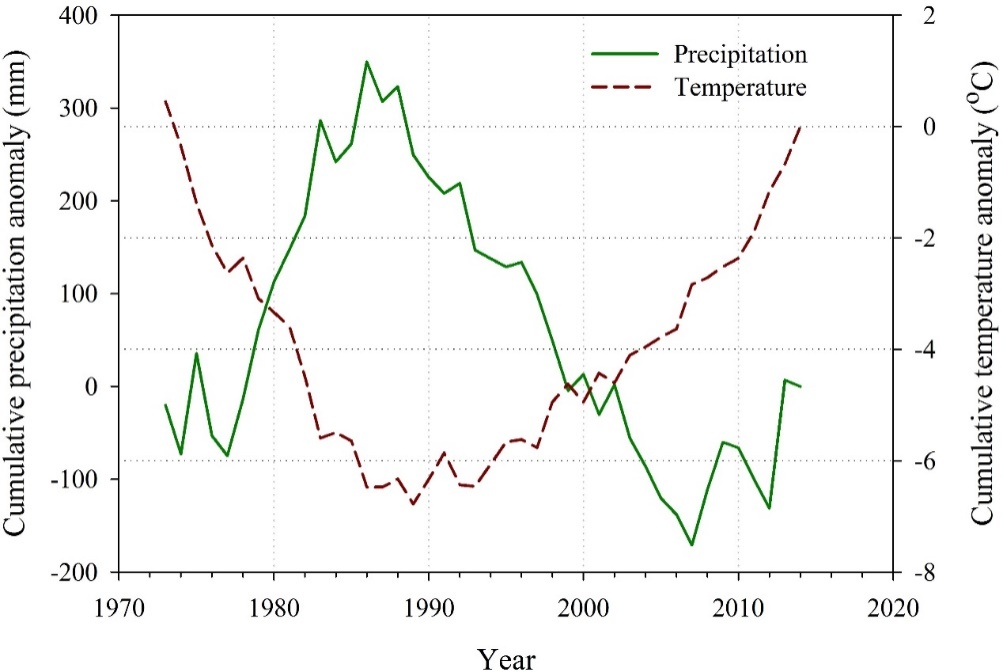


**Figure S3.**The cumulative summer tempertature (June-September) and annual total precipitation anomalies recorded at Burang station, showing the abupt shifts of both summer temprature and annual total precipitation in the late-1980s.

**
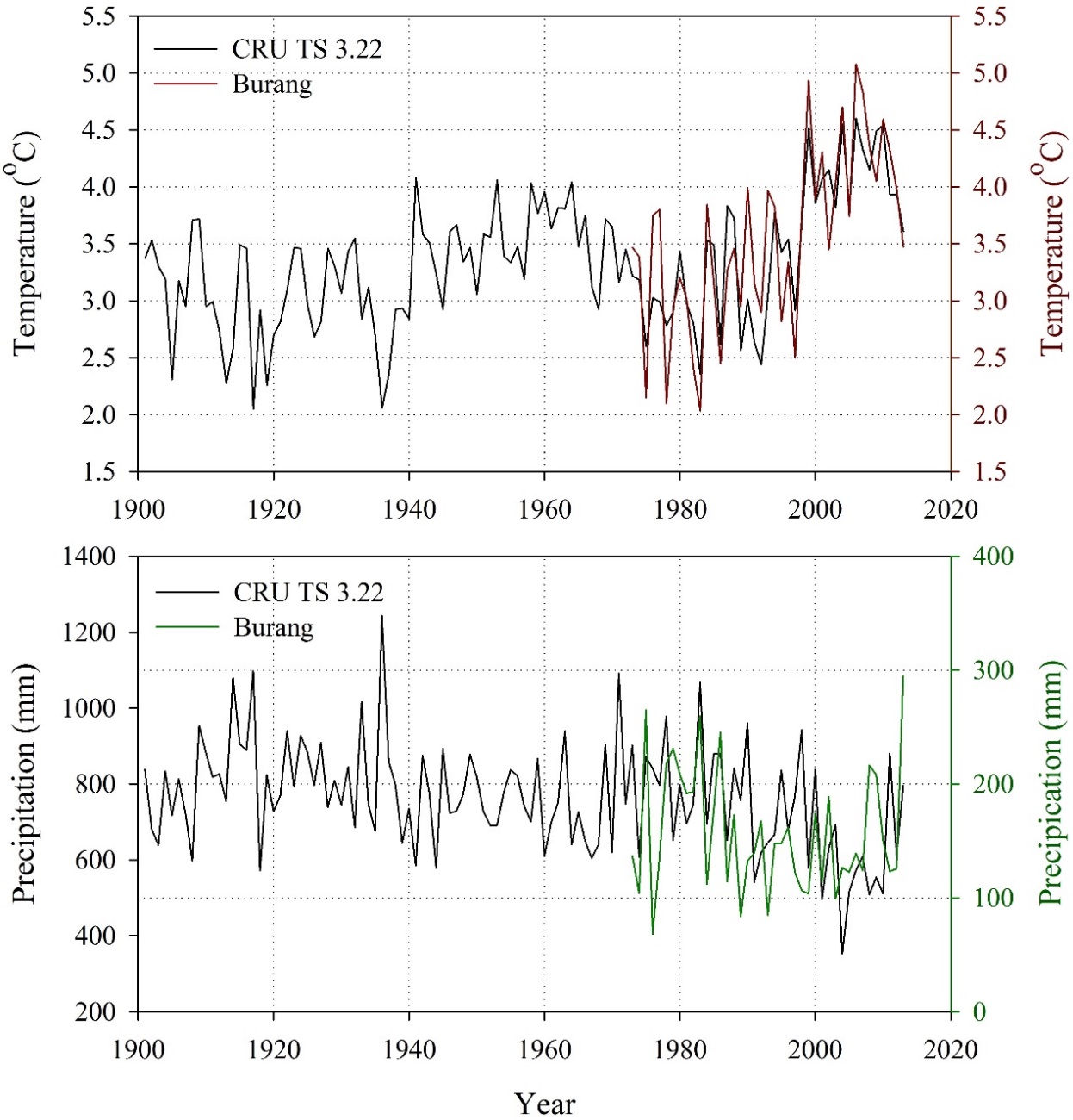
**

**Figure S4.** The annaul temperature and preciptation variations at Burang (1973-2014) and at the grid covering Burang station from the CRU TS 3.22 dataset (1901-2013).

**
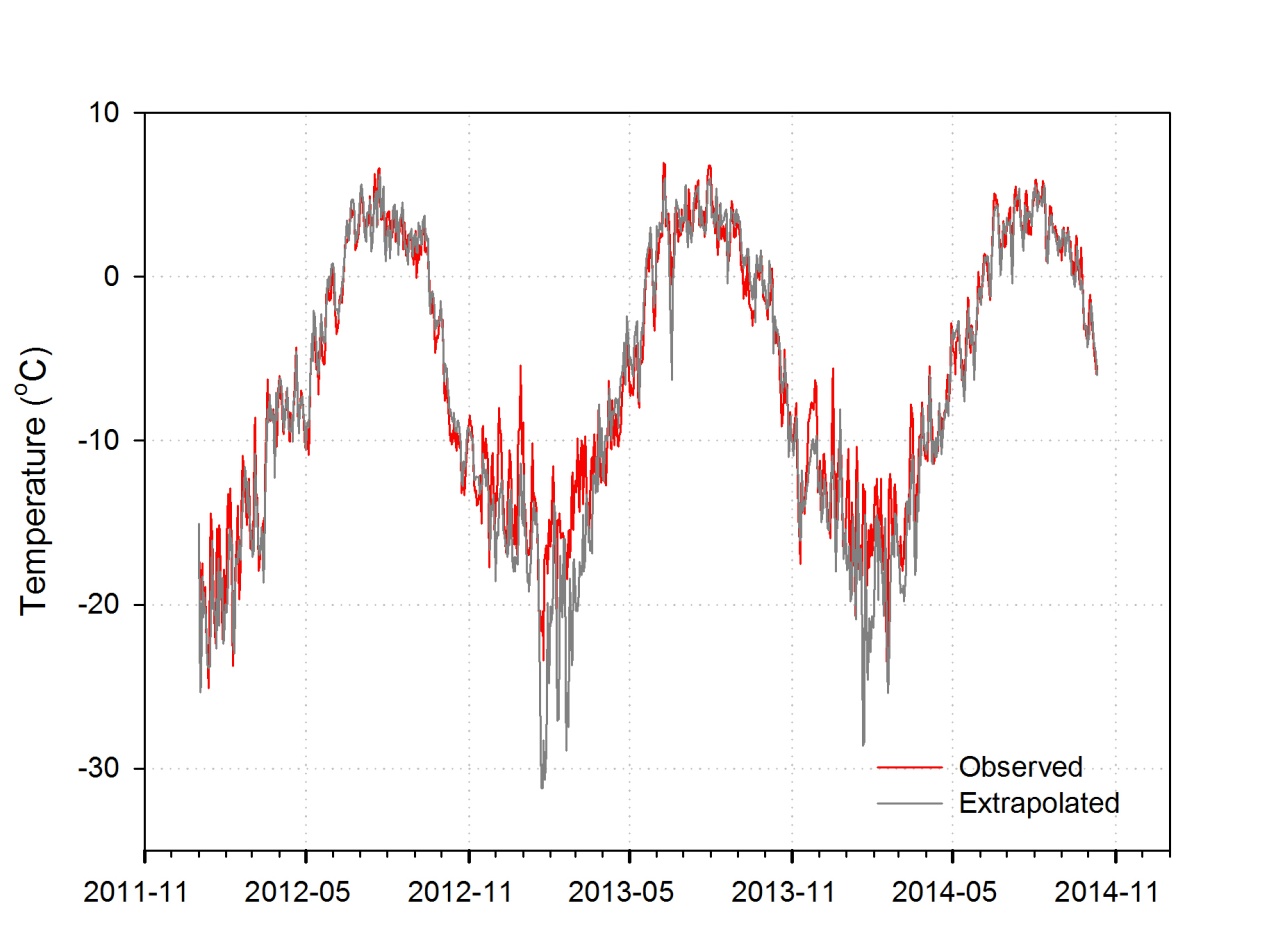
**

**Figure S5.** The observed and extrapolated air temperature at the AWS1 at the termiuns of Naimona’nyi Glacier during the period from January 2012 to October 2014.
